# Supplementary material for: Hippocampal microstructure, but not macrostructure, mediates age differences in episodic memory
Source: Front Aging Neurosci. 2023 Nov 20;15:1285375. doi: 10.3389/fnagi.2023.1285375 (PMC10694359; doi:10.3389/fnagi.2023.1285375)
Supplement: Supplementary file 1 [file Data_Sheet_1.docx]

**Supplementary Table 1.** Correlation coefficients for measured variables in younger adults

|  | Age | 1 | 2 | 3 | 4 | 5 | 6 | 7 | 8 | 9 | 10 |
| --- | --- | --- | --- | --- | --- | --- | --- | --- | --- | --- | --- |
| 1. Intracellular R | **-0.26** |  |  |  |  |  |  |  |  |  |  |
| 2. Intracellular L | -0.23 | **0.86** |  |  |  |  |  |  |  |  |  |
| 3. Dispersion R | -0.08 | **0.66** | **0.52** |  |  |  |  |  |  |  |  |
| 4. Dispersion L | -0.15 | **0.60** | **0.60** | **0.74** |  |  |  |  |  |  |  |
| 5. Free R | -0.13 | **0.83** | **0.72** | **0.60** | **0.60** |  |  |  |  |  |  |
| 6. Free L | -0.16 | **0.72** | **0.86** | **0.49** | **0.60** | **0.78** |  |  |  |  |  |
| 7. Volume R | 0.14 | -0.08 | -0.03 | 0.05 | -0.01 | -0.08 | -0.05 |  |  |  |  |
| 8. Volume L | 0.14 | -0.06 | -0.20 | 0.05 | -0.01 | 0.06 | -0.18 | **0.60** |  |  |  |
| 9. Recognition | -0.12 | 0.13 | 0.21 | 0.12 | 0.22 | 0.15 | 0.23 | -0.11 | -0.11 |  |  |
| 10. Immediate recall | -0.04 | 0.20 | 0.26 | 0.10 | 0.18 | 0.19 | 0.20 | -0.01 | -0.09 | **0.57** |  |
| 11. Delayed recall | -0.05 | 0.22 | **0.32** | 0.11 | 0.23 | 0.22 | 0.25 | -0.03 | -0.13 | **0.61** | **0.80** |

*Notes.* Correlation coefficients are shown for comparisons among age, hippocampal diffusion and volume from each hemisphere (L = left, R = right), and memory performance in younger adults. Significant effects survived Bonferroni correction for 11 comparisons per measure (bolded; *p* < 0.0045).

**Supplementary Table 2.** Correlation coefficients for measured variables in older adults

|  | Age | 1 | 2 | 3 | 4 | 5 | 6 | 7 | 8 | 9 | 10 |
| --- | --- | --- | --- | --- | --- | --- | --- | --- | --- | --- | --- |
| 1. Intracellular R | 0.12 |  |  |  |  |  |  |  |  |  |  |
| 2. Intracellular L | 0.09 | **0.82** |  |  |  |  |  |  |  |  |  |
| 3. Dispersion R | 0.11 | **0.66** | **0.62** |  |  |  |  |  |  |  |  |
| 4. Dispersion L | 0.03 | **0.48** | **0.68** | **0.73** |  |  |  |  |  |  |  |
| 5. Free R | -0.06 | **0.84** | **0.71** | **0.60** | **0.42** |  |  |  |  |  |  |
| 6. Free L | -0.05 | **0.73** | **0.84** | **0.55** | **0.61** | **0.78** |  |  |  |  |  |
| 7. Volume R | -0.21 | -0.29 | -0.16 | -0.04 | 0.05 | -0.21 | -0.14 |  |  |  |  |
| 8. Volume L | -0.33 | -0.15 | -0.10 | -0.06 | 0.21 | -0.08 | -0.03 | **0.69** |  |  |  |
| 9. Recognition | -0.002 | **0.39** | **0.37** | **0.40** | 0.33 | 0.22 | 0.33 | -0.06 | -0.01 |  |  |
| 10. Immediate recall | 0.10 | **0.38** | **0.40** | 0.28 | 0.24 | 0.25 | 0.34 | -0.13 | -0.13 | **0.51** |  |
| 11. Delayed recall | 0.07 | **0.37** | **0.48** | 0.32 | 0.37 | 0.29 | **0.44** | -0.05 | -0.004 | **0.61** | **0.81** |

*Notes.* Correlation coefficients are shown for comparisons among age, hippocampal diffusion and volume from each hemisphere (L = left, R = right), and memory performance in older adults. Significant effects survived Bonferroni correction for 11 comparisons per measure (bolded; *p* < 0.0045).

**Supplementary Table 3.** Confirmatory factor analysis model fits

| Fit Estimates | First-Order Model | Second-Order Model |
| --- | --- | --- |
| Chi-square (df), *p* | 159.4 (34), *p* < .001 | 188.5 (38), *p* < .001 |
| RMSEA | 0.16 | 0.17 |
| CFI | **0.91** | **0.90** |
| SRMR | **0.03** | **0.07** |

*Notes.* Model fit estimates are bolded if significant at thresholds reported in the Methods section. RMSEA = root mean square error of approximation. CFI = comparative fit index. SRMR = standardized root mean residual.

**Supplementary Table 4.** Model of covariances model fits

|  | Covariance-Only |
| --- | --- |
| *Fit estimates* |  |
| Chi-square (df), *p* | 224.3 (46), *p* < .001 |
| RMSEA | 0.17 |
| CFI | 0.89 |
| SRMR | **0.07** |

*Notes.* Model fit estimates are bolded if significant at thresholds reported in the Methods section. A Chi-square difference test revealed that the original model in all participants provided a significantly better fit than this covariance-only model, *p* < 0.001. RMSEA = root mean square error of approximation. CFI = comparative fit index. SRMR = standardized root mean residual.

**Supplementary Table 5.** Standardized and unstandardized path estimates with confidence intervals

|  | All Participants | Older Adults |
| --- | --- | --- |
| *Standardized path estimates* |  |  |
| Total effect | **-0.44 (-0.57, -0.29)** | 0.07 (-0.20, 0.34) |
| Total indirect effect | **-0.24 (-0.43, -0.06)** | 0.03 (-0.16, 0.18) |
| Diffusion indirect effect | **-0.26 (n/a)** | 0.04 (n/a) |
| Volume indirect effect | 0.02 (n/a) | <0.01 (n/a) |
| Influential pathway indirect effect | <0.01 (n/a) | <0.01 (n/a) |
| Age to memory direct effect | -0.20 (-0.42, 0.04) | 0.04 (-0.20, 0.31) |
| *Unstandardized path estimates* |  |  |
| Total effect | **-0.04 (-0.06, -0.03)** | 0.03 (-0.09, 0.16) |
| Total indirect effect | **-0.02 (-0.04, -0.006)** | 0.01 (-0.07, 0.09) |
| Diffusion indirect effect | **-0.03 (-0.04, -0.02)** | 0.02 (-0.04, 0.09) |
| Volume indirect effect | 0.002 (-0.01, 0.01) | -0.006 (-0.07, 0.03) |
| Influential pathway indirect effect | <0.001 (-0.004, 0.001) | <0.001 (-0.01, 0.002) |
| Age to memory direct effect | -0.02 (-0.04, 0.003) | 0.02 (-0.10, 0.14) |

*Notes.* Standardized and unstandardized path estimates with 95% confidence intervals (lower limit, upper limit) are presented for the original model in all participants (left column) and the exploratory original model in older adults only (right column).

**Supplementary Figure 1.** First-order (A) and second-order (B) confirmatory factor analysis models and a model of covariances (C). Circles represent latent variables, rectangles represent measured variables, and double-headed curved arrows indicate covariances. Bolded values depict significant correlations between latent variables. L/R = left/right hemisphere.
